# Nursing education action in the quality of life of patients with diabetes mellitus type I

**DOI:** 10.1186/1758-5996-7-S1-A169

**Published:** 2015-11-11

**Authors:** Carla Renata Correa Porto, Laura Lippi Gonçalves, Izabel Cristina Ribeiro da Silva Saccomann

**Affiliations:** 1 Pontificia Universidade Católica de São Paulo, São Paulo, Brazil

## Background

The measurement of quality of life (QOL) in patients with diabetes and its complications has been the subject of several studies in order to determine the necessary changes to improve their welfare. Thus, health education plays a significant role in the diabetic patient's therapy.

## Objectives

Apply an educative action in relation to medication and non-medication treatment for patients with diabetes type 1.

## Materials and methods

An exploratory, descriptive and quasi-experimental study in outpatients with Diabetes Type I whose data collection was conducted in two phases. In the first phase the educational action was held during seven meetings and in the second phase, three months after, questionnaires on patient satisfaction and evaluation of quality of life were applied. The educational action focused on the beliefs of self-efficacy for medication and non-medication treatment aiming at changing behavior for self-care and consequent improvement of QOL. It was used Diabetes Quality of Life Measure (DQOL) and patient satisfaction instrument.

## Results

The sample included 23 patients with DM type I, the majority were women (70%), and the average age was 34 yrs. old (±10.17). After the educational intervention, there was a decrease in QOL scores, in which men showed to be less concerned about than diabetes women; patients who held a job were more satisfied with their quality of life and the disease had less impact on their life; and those who were not used to drinking alcohol were more concerned with the disease. Regarding the educational action, patients reported that it had contributed to food consumption, improved the quality of life and the glycemic control, and clarified the use of insulin and foot care.

## Conclusion

The proposed educational action was considered by most patients as excellent and satisfactory, and to have contributed to the disease control. Therefore, it is vital for the health team intervention proposals that are effective for self-care and as a consequence an improvement in the disease management and in the quality of life.

